# Publisher Correction: The cooperative action of CSB, CSA, and UVSSA target TFIIH to DNA damage-stalled RNA polymerase II

**DOI:** 10.1038/s41467-020-19643-7

**Published:** 2020-11-06

**Authors:** Yana van der Weegen, Hadar Golan-Berman, Tycho E. T. Mevissen, Katja Apelt, Román González-Prieto, Joachim Goedhart, Elisheva E. Heilbrun, Alfred C. O. Vertegaal, Diana van den Heuvel, Johannes C. Walter, Sheera Adar, Martijn S. Luijsterburg

**Affiliations:** 1 Department of Human Genetics, Leiden University Medical Center, Einthovenweg 20, 2333 ZC Leiden, the Netherlands; 2 Department of Microbiology and Molecular Genetics, The Institute for Medical Research Israel–Canada, The Faculty of Medicine, The Hebrew University of Jerusalem, Ein Kerem, Jerusalem, 91120 Israel; 3 Howard Hughes Medical Institute and Department of Biological Chemistry and Molecular Pharmacology, Harvard Medical School, Boston, MA 02115 USA; 4 Department of Cell and Chemical Biology, Leiden University Medical Center, Einthovenweg 20, 2333 ZC Leiden, the Netherlands; 5 Swammerdam Institute for Life Sciences, Section of Molecular Cytology, van Leeuwenhoek Centre for Advanced Microscopy, University of Amsterdam, Science Park 904, 1098 XH Amsterdam, the Netherlands

**Keywords:** DNA damage and repair, Nucleotide excision repair, Transcription

Correction to: *Nature Communications* 10.1038/s41467-020-15903-8, published online 30 April 2020.

The original version of this Article contained an error in the hyperlink to an interactive volcano plot for Figure 5 panel b (GFP-UVSSA vs GFP-UVSSA+UV). This has been corrected in both the PDF and HTML versions of the Article.

