# Supplementary material for: The cooperative action of CSB, CSA, and UVSSA target TFIIH to DNA damage-stalled RNA polymerase II
Source: Nat Commun. 2020 Apr 30;11:2104. doi: 10.1038/s41467-020-15903-8 (PMC7192910; doi:10.1038/s41467-020-15903-8)
Supplement: Supplementary file 1 — Supplementary Information [file 41467_2020_15903_MOESM1_ESM.pdf]

Supplementary Information

The cooperative action of CSB, CSA and UVSSA target TFIIH to DNA damage-stalled RNA polymerase II

Yana van der Weegen, Hadar Golan Berman, Tycho E.T. Mevissen, Katja Apelt, Román González-Prieto, Joachim Goedhart, Elisheva E. Heilbrun, Alfred C.O. Vertegaal, Diana van den Heuvel, Johannes C. Walter, Sheera Adar, and Martijn S. Luijsterburg*

* Corresponding author: Martijn Luijsterburg (m.luijsterburg@lumc.nl)

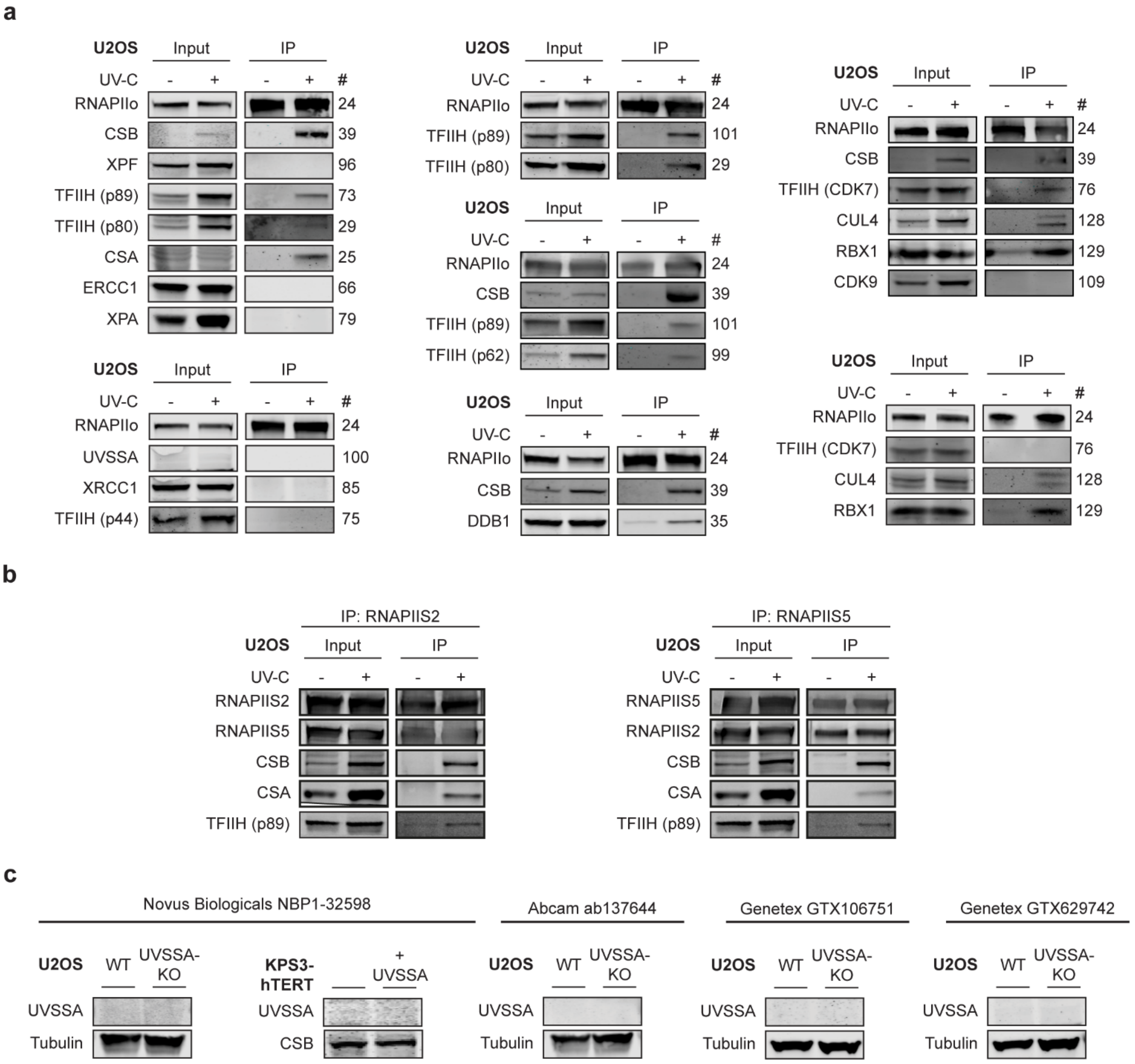
a
U2OS
Input
IP
UV-C
-
+
#
RNAPIIo
CSB
XPF
TFIIH (p89)
TFIIH (p80)
CSA
ERCC1
XPA
24
39
96
73
29
25
66
79
UVSSA
XRCC1
TFIIH (p44)
100
85
75
TFIIH (p62)
101
99
DDB1
35
TFIIH (CDK7)
CUL4
RBX1
CDK9
76
128
129
109
b
IP: RNAPIIS2
IP: RNAPIIS5
RNAPIIS2
RNAPIIS5
c
Novus Biologicals NBP1-32598
Abcam ab137644
Genetex GTX106751
Genetex GTX629742
WT
UVSSA-KO
KPS3-hTERT
+ UVSSA
Tubulin

Supplementary Figure 1. Testing of antibodies in IP and whole cell lysates. (a) Multiple endogenous RNAPII Co-IP experiments in WT cells for staining with various antibodies. **Figure 1b** is composed of these Co-IPs. We were not always able to detect CDK7 probably due to weak interactions or inefficient antibody. (b) Endogenous RNAPII Co-IP using either a Ser2-phosphorylated RNAPII antibody or a Ser5-phosphorylated RNAPII antibody. (c) Testing of various UVSSA antibodies in KPS3-hTERT, KPS3-hTERT + UVSSA, U2OS (FRT) WT, and U2OS (FRT) UVSSA-KO cells. At least two independent replicates of each IP experiment were performed obtaining similar results. Source data are provided as a Source Data file.

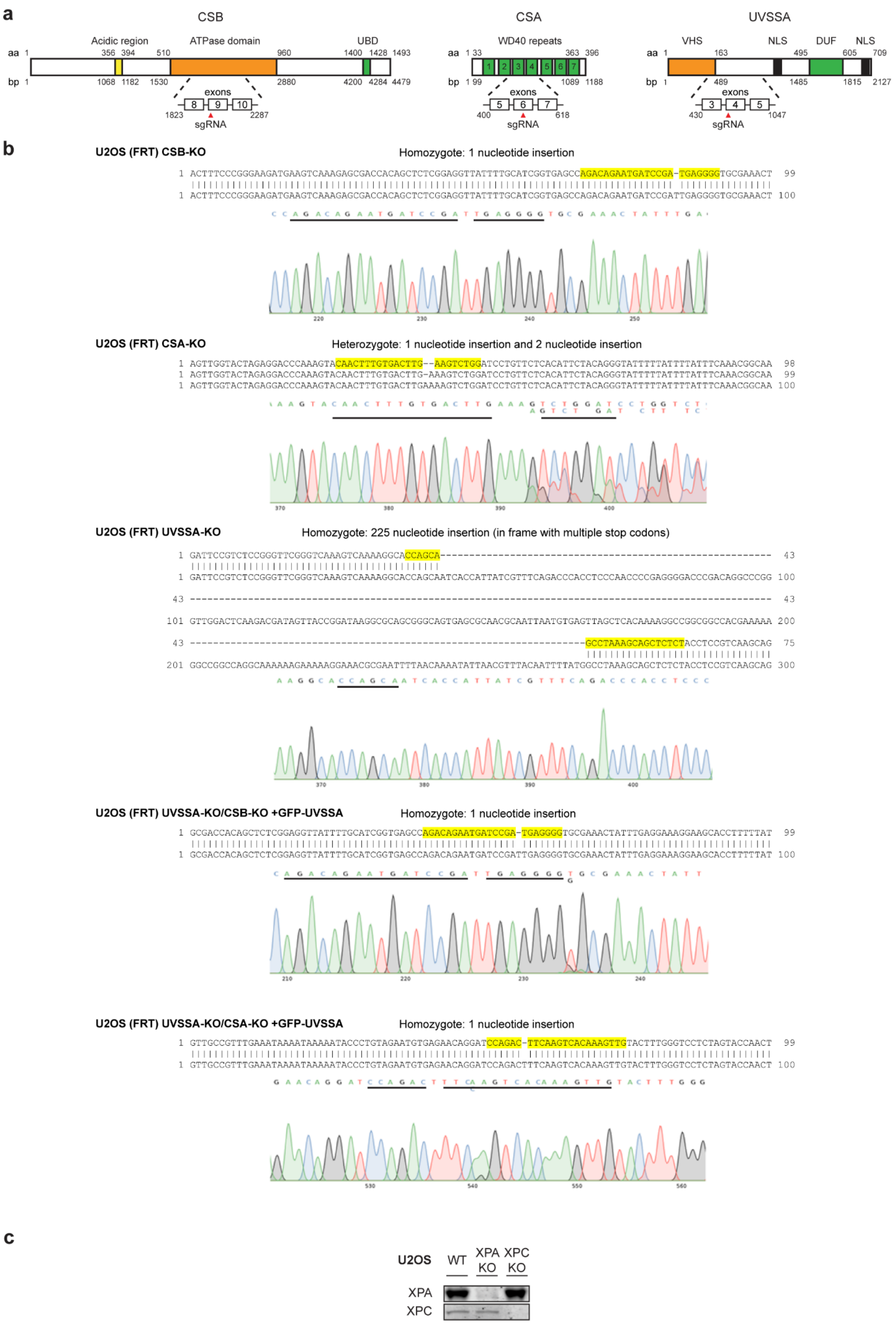
a
CSB
Acidic region
ATPase domain
UBD
aa 1 356 394 510 960 1400 1428 1493
bp 1 1068 1182 1530 2880 4200 4284 4479
exons
8 9 10
1823 2287
sgRNA
CSA
WD40 repeats
aa 1 33 363 396
1 2 3 4 5 6 7
bp 1 99 1089 1188
exons
5 6 7
400 618
sgRNA
UVSSA
VHS
NLS
DUF
NLS
aa 1 163 495 605 709
bp 1 489 1485 1815 2127
exons
3 4 5
430 1047
sgRNA
b
U2OS (FRT) CSB-KO
Homozygote: 1 nucleotide insertion
1 ACTTTCCCGGGAAGATGAAGTCAAAGAGCGACCACAGCTCTCGGAGGTTATTTTGCATCGGTGAGCCAGACAGAATGATCCGA-TGAGGGGTGCGAAACT 99
1 ACTTTCCCGGGAAGATGAAGTCAAAGAGCGACCACAGCTCTCGGAGGTTATTTTGCATCGGTGAGCCAGACAGAATGATCCGATTGAGGGGTGCGAAACT 100
220 230 240 250
U2OS (FRT) CSA-KO
Heterozygote: 1 nucleotide insertion and 2 nucleotide insertion
1 AGTTGGTACTAGAGGACCCAAAGTACAACTTTGTGACTTG--AAGTCTGGATCCTGTTCTCACATTCTACAGGGTATTTTTATTTATTTCAAACGGCAA 98
1 AGTTGGTACTAGAGGACCCAAAGTACAACTTTGTGACTTG-AAAGTCTGGATCCTGTTCTCACATTCTACAGGGTATTTTTATTTATTTCAAACGGCAA 99
1 AGTTGGTACTAGAGGACCCAAAGTACAACTTTGTGACTTGAAAAGTCTGGATCCTGTTCTCACATTCTACAGGGTATTTTTATTTATTTCAAACGGCAA 100
370 380 390 400
U2OS (FRT) UVSSA-KO
Homozygote: 225 nucleotide insertion (in frame with multiple stop codons)
1 GATTCCGTCTCCGGGTTCGGGTCAAAGTCAAAAGGCACCAGCA 43
1 GATTCCGTCTCCGGGTTCGGGTCAAAGTCAAAAGGCACCAGCAATCACCATTATCGTTTCAGACCCACCTCCCAACCCCGAGGGGACCCGACAGGCCCGG 100
43 43
101 GTTGGACTCAAGACGATAGTTACCGGATAAGGCGCAGCGGGCAGTGAGCGCAACGCAATTAATGTGAGTTAGCTCACAAAAGGCCGGCGGCCACGAAAAA 200
43 GCCTAAAGCAGCTCTCTACCTCCGTCAAGCAG 75
201 GGCCGGCCAGGCAAAAAAGAAAAAGGAAACGCGAATTTTAACAAAATATTAACGTTTACAATTTTATGCCTAAAGCAGCTCTCTACCTCCGTCAAGCAG 300
370 380 390 400
U2OS (FRT) UVSSA-KO/CSB-KO +GFP-UVSSA
Homozygote: 1 nucleotide insertion
1 GCGACCACAGCTCTCGGAGGTTATTTTGCATCGGTGAGCCAGACAGAATGATCCGA-TGAGGGGTGCGAAACTATTTGAGGAAAGGAAGCACCTTTTTAT 99
1 GCGACCACAGCTCTCGGAGGTTATTTTGCATCGGTGAGCCAGACAGAATGATCCGATTGAGGGGTGCGAAACTATTTGAGGAAAGGAAGCACCTTTTTAT 100
210 220 230 240
U2OS (FRT) UVSSA-KO/CSA-KO +GFP-UVSSA
Homozygote: 1 nucleotide insertion
1 GTTGCCGTTTGAAATAAAATAAAAATACCCTGTAGAATGTGAGAACAGGATCCAGAC-TTCAAGTCACAAAGTTGTACTTTGGGTCCTCTAGTACCAACT 99
1 GTTGCCGTTTGAAATAAAATAAAAATACCCTGTAGAATGTGAGAACAGGATCCAGACTTTCAAGTCACAAAGTTGTACTTTGGGTCCTCTAGTACCAACT 100
530 540 550 560
c
U2OS
WT
XPA KO
XPC KO
XPA
XPC

Supplementary Figure 2. **Sequence of CSB, CSA, and UVSSA-KO cells**. (a) A schematic representation of CSB, CSA, and UVSSA including the location of the guide RNAs used for the generation of the CRISPR/Cas9-mediated KO. (b) Sequences of CSB, CSA, and UVSSA knockouts. (c) Western blot analysis of XPC and XPA knockouts (n=2). Source data are provided as a Source Data file.

a

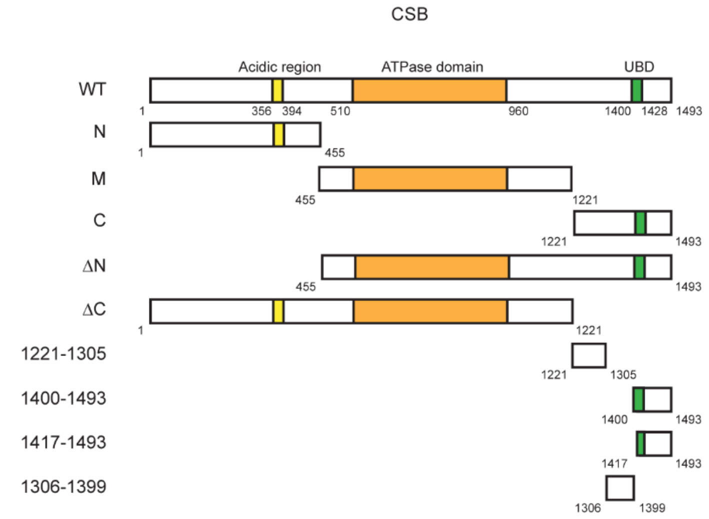
CSB
Acidic region
ATPase domain
UBD
WT
N
M
C
ΔN
ΔC
1221-1305
1400-1493
1417-1493
1306-1399
1
356
394
510
960
1400
1428
1493
455
1221
1305
1417
1306
1399

b

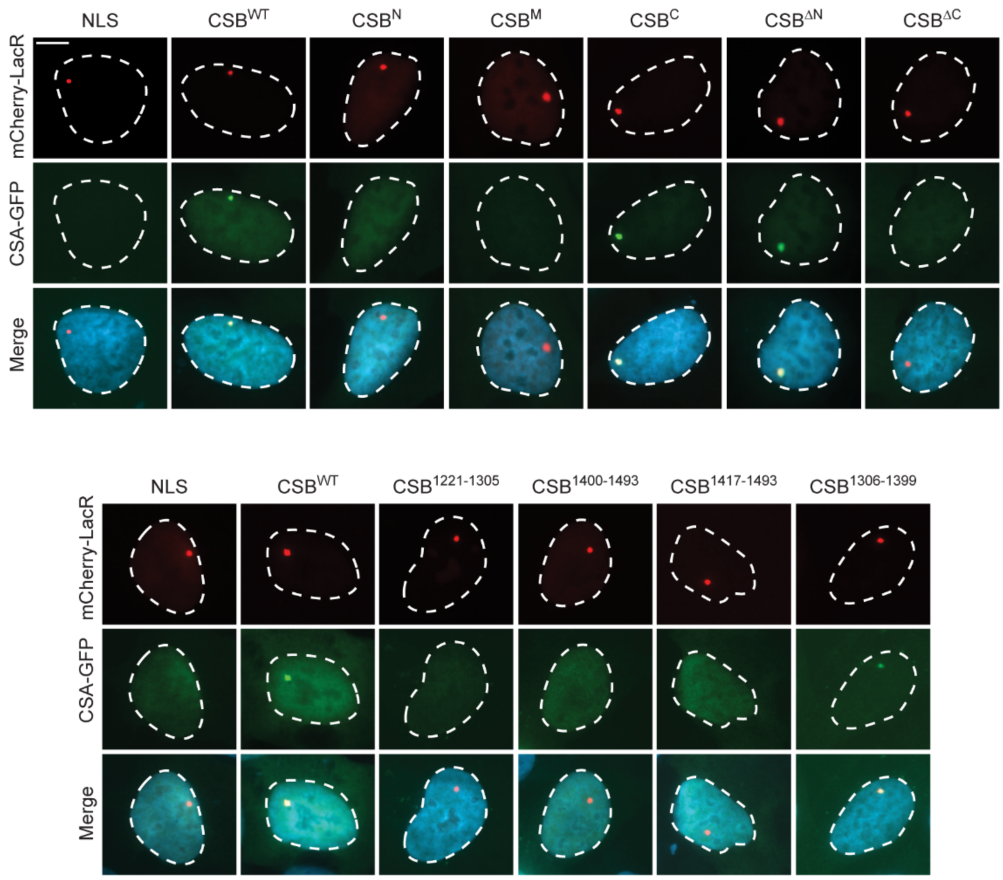
NLS
CSB^WT
CSB^N
CSB^M
CSB^C
CSB^ΔN
CSB^ΔC
mCherry-LacR
CSA-GFP
Merge
CSB^1221-1305
CSB^1400-1493
CSB^1417-1493
CSB^1306-1399

c

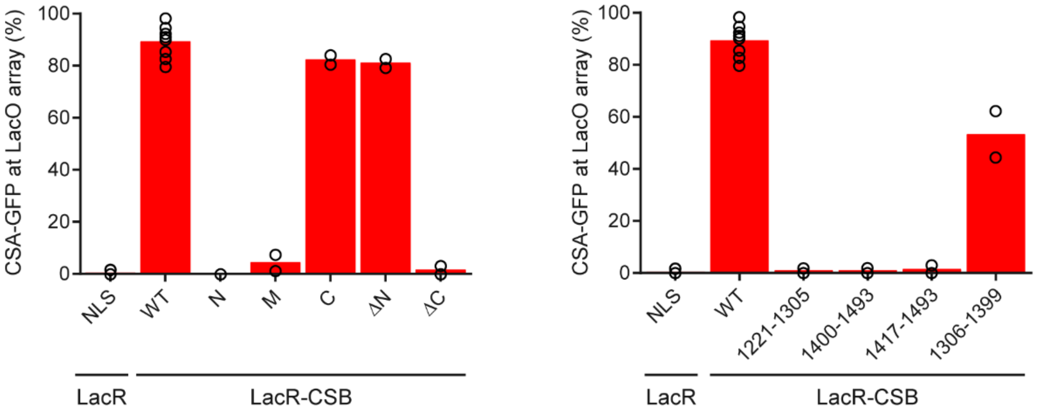
CSA-GFP at LacO array (%)
0
20
40
60
80
100
NLS
WT
N
M
C
ΔN
ΔC
1221-1305
1400-1493
1417-1493
1306-1399
LacR
LacR-CSB

Supplementary Figure 3. CSA interacts with the C-terminal region of CSB. (a) A schematic representation of CSB and its deletion mutants. (b) Recruitment of CSA-GFP to the LacO array upon tethering of the indicated mCherry-LacR fusion proteins (scale bar = 5 μm). (c) Quantification of CSA-GFP and mCherry-LacR-CSB co-localization at the LacO array. Each symbol represents the mean of an independent experiment (n=2 for all except LacR-NLS and LacR-CSB^{WT} which is n=8, >50 cells collected per experiment). Source data are provided as a Source Data file.

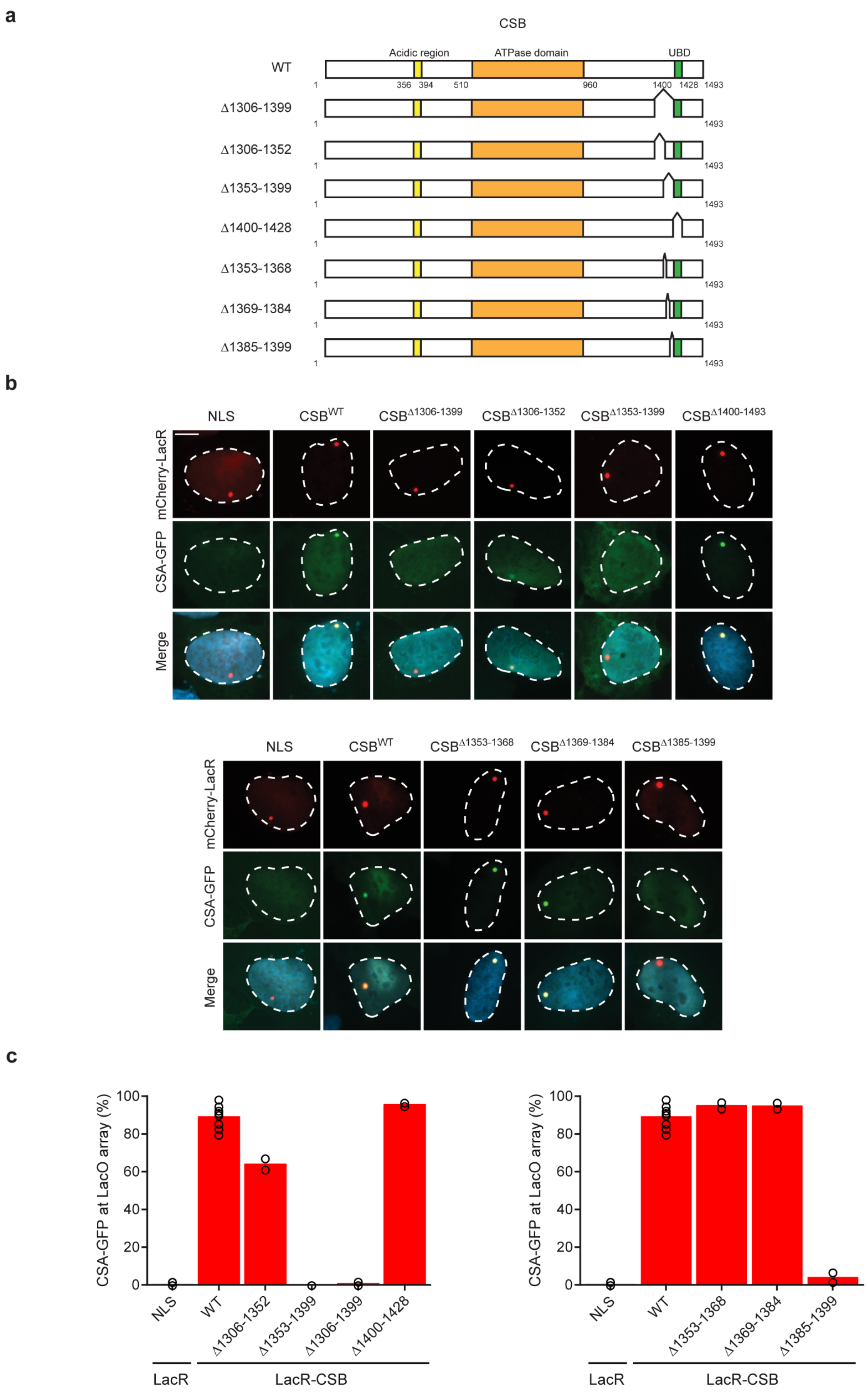

a
CSB
Acidic region
ATPase domain
UBD
WT
Δ1306-1399
Δ1306-1352
Δ1353-1399
Δ1400-1428
Δ1353-1368
Δ1369-1384
Δ1385-1399
1
356
394
510
960
1400
1428
1493
b
NLS
CSB^WT
CSB^Δ1306-1399
CSB^Δ1306-1352
CSB^Δ1353-1399
CSB^Δ1400-1493
mCherry-LacR
CSA-GFP
Merge
CSB^Δ1353-1368
CSB^Δ1369-1384
CSB^Δ1385-1399
c
CSA-GFP at LacO array (%)
0
20
40
60
80
100
NLS
WT
Δ1306-1352
Δ1353-1399
Δ1306-1399
Δ1400-1428
Δ1353-1368
Δ1369-1384
Δ1385-1399
LacR
LacR-CSB

Supplementary Figure 4. CSA interacts with amino acids 1385-1399 of CSB. (a) A schematic representation of CSB and its deletion mutants. (b) Recruitment of CSA-GFP to the LacO array upon tethering of the indicated mCherry-LacR fusion proteins (scale bar = 5 μm). (c) Quantification of CSA-GFP and mCherry-LacR-CSB co-localization at the LacO array. Each symbol represents the mean of an independent experiment (n=2 for all except LacR-NLS and LacR-CSB^{WT} which is n=8, >50 cells collected per experiment). Source data are provided as a Source Data file.

a

		CIM	
1493	sp|Q03468|ERCC6_HUMAN	ASSSLLAKMRARNHL	Homo sapiens (Human)
1491	tr|H2Q1W1|H2Q1W1_PANTR	ASSSLLAKMRARNHL	Pan troglodytes (Chimpanzee)
1493	tr|G3QVF5|G3QVF5_GORGO	ASSSLLAKMRARNHL	Gorilla gorilla gorilla (Western lowland gorilla)
1492	tr|G1S127|G1S127_NOMLE	ASSSLLAKMRARNHL	Nomascus leucogenys (Northern white-cheeked gibbon)
1491	tr|A0A2R8ZA95|A0A2R8ZA95_PANPA	ASSSLLAKMRARNHL	Pan paniscus Bonobo)
1482	tr|E1BFL2|E1BFL2_BOVIN	TSSSLLAKMRARNHL	Bos taurus (Bovine)
1484	tr|M3XEB4|M3XEB4_FELCA	PSSSLLAKMRARNHL	Felis catus (Cat)
1486	tr|E2QSK6|E2QSK6_CANLF	PSSSLLAKMRARNHL	Canis lupus familiaris (Dog)
1460	tr|F7D5S6|F7D5S6_HORSE	TSSSLLAKMRARNHL	Equus caballus (Horse)
1432	tr|G3TCV9|G3TCV9_LOXAF	TSSSLLAKMRARNHL	Loxodonta africana (African elephant)
1481	tr|A0A0P6J577|A0A0P6J577_HETGA	TSSSLLARMRARNHL	Heterocephalus glaber (Naked mole rat)
1481	tr|F8VPZ5|F8VPZ5_MOUSE	SSSSLLARMRARNHM	Mus musculus (Mouse)
1485	tr|A0A2Y9MEF2|A0A2Y9MEF2_DELLE	TSSSLLAKMRARNHL	Delphinapterus leucas (Beluga whale)
1462	tr|A0A2U4AKE1|A0A2U4AKE1_TURTR	TSSSLLAKMRARNHL	Tursiops truncatus (Atlantic bottle-nosed dolphin)
1488	tr|A0A1S3N477|A0A1S3N477_SALSA	SSSTLLARMKARNYL	Salmo salar (Atlantic salmon)
1409	tr|A0A2D0QRA6|A0A2D0QRA6_ICTPU	SSSSLLARMRARNHV	Ictalurus punctatus (Channel catfish)
1389	tr|F1R294|F1R294_DANRE	SSSSLLARMRARNHL	Danio rerio (Zebrafish)
1370	tr|A0A1L8FKT9|A0A1L8FKT9_XENLA	SSSSLLARMKARNNL	Xenopus laevis (African clawed frog)
1387	tr|H3AWF0|H3AWF0_LATCH	SSSSLLANMRARNHL	Latimeria chalumnae (West Indian ocean coelacanth)
1187	sp|Q9ZV43|CHR8_ARATH	SSAELLNRIRGSREQ	Arabidopsis thaliana (Mouse-ear cress)
1015	tr|A8XNA8|A8XNA8_CAEBR	---------------	Caenorhabditis briggsae (nematode)
957	tr|Q93781|Q93781_CAEEL	---------------	Caenorhabditis elegans (nematode)
973	sp|Q9UR24|RHP26_SCHPO	---TLLARLKQRR--	Schizosaccharomyces pombe (Fission yeast)
1085	sp|P40352|RAD26_YEAST	NYDDGIT-FA--RSK	Saccharomyces cerevisiae (Baker's yeast)
1037	tr|W0T437|W0T437_KLUMD	LKVKTLPSQE--KKK	Kluyveromyces marxianus (Yeast)
925	tr|Q6WD94|Q6WD94_GIAIN	---------------	Giardia intestinalis (intestinal parasite)
1222	tr|A0A1V9XZ12|A0A1V9XZ12_9ACAR	SPSRPKGKRRSVAVL	Tropilaelaps mercedesae (bee mite)
1125	tr|A0A2A3EJZ4|A0A2A3EJZ4_APICC	---------------	Apis cerana cerana (Oriental honeybee)
1073	tr|A0A026W8Z2|A0A026W8Z2_OOCBI	---------------	Ooceraea biroi (Clonal raider ant)
1005	tr|E2BDE2|E2BDE2_HARSA	---------------	Harpegnathos saltator (Jerdon's jumping ant)

b

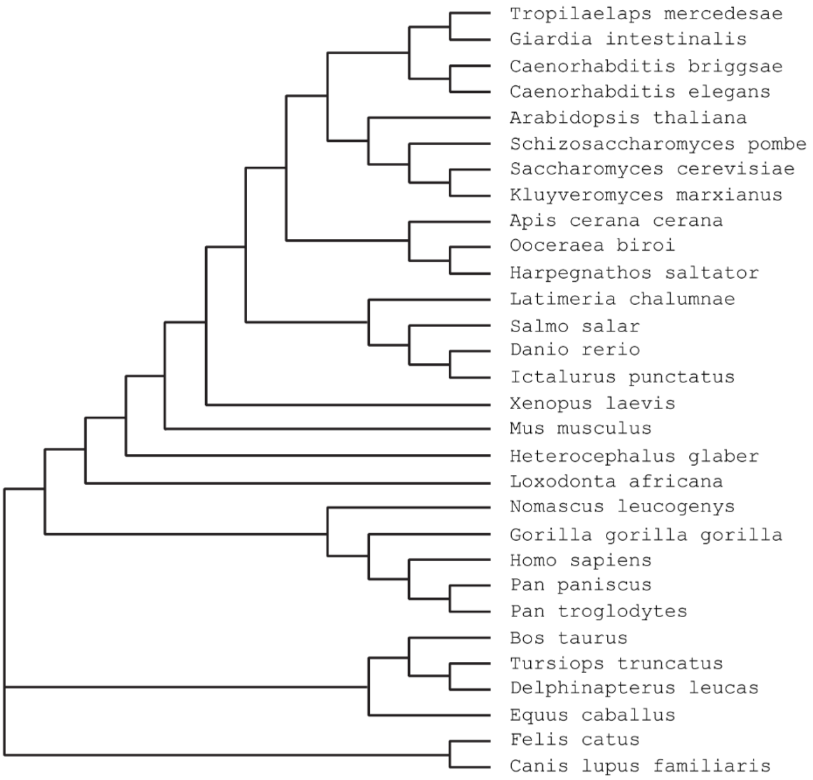

Supplementary Figure 5. Alignment of CSB and CSB orthologues. (a) Alignment of the C-terminal CIM of CSB orthologues from a variety of different species. Sequences were aligned with ClustalW (b) A phylogenetic tree was constructed based on the alignment of CSB orthologues using ClustalW.

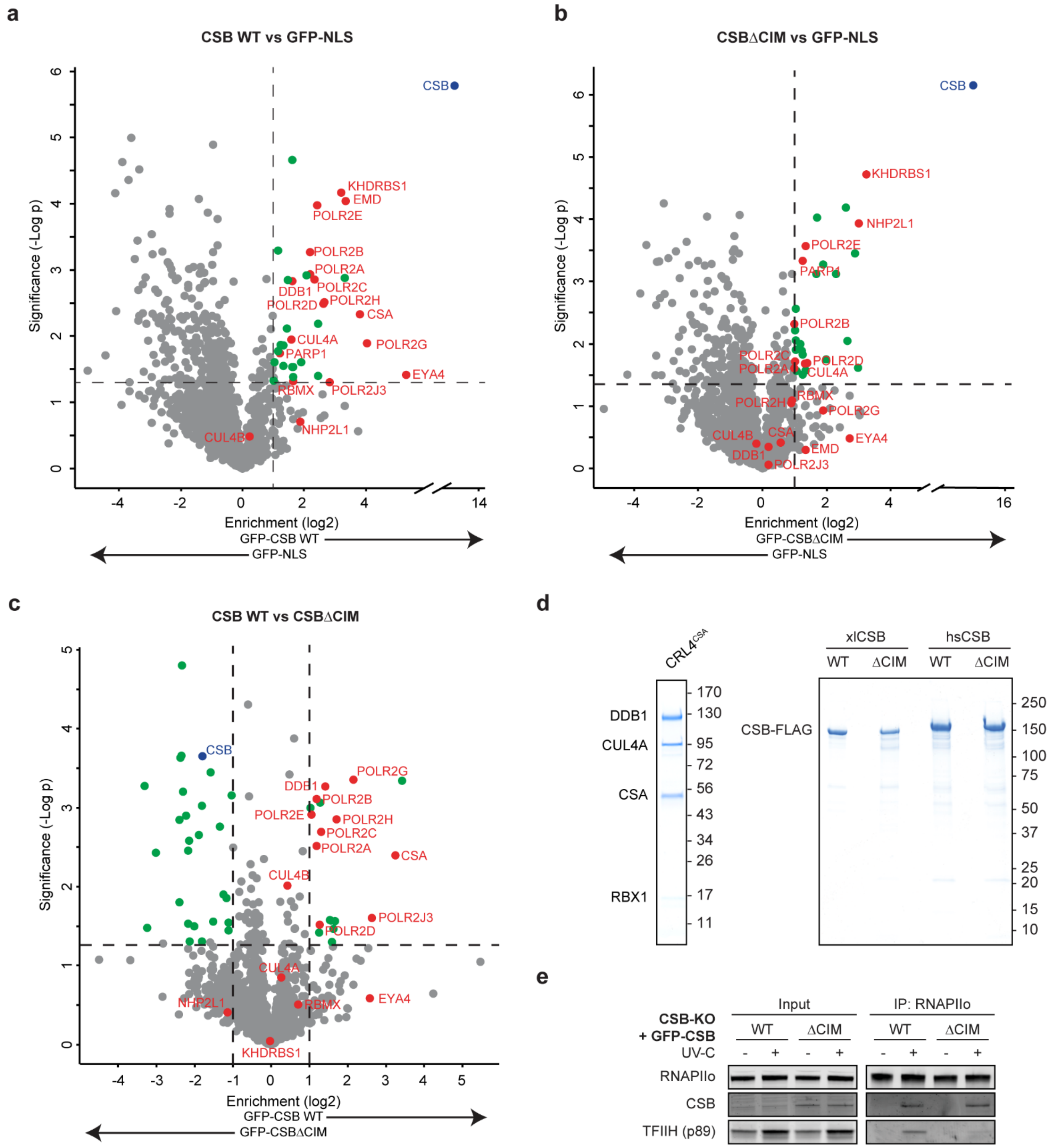
a
CSB WT vs GFP-NLS
Significance (-Log p)
Enrichment (log2)
GFP-CSB WT
GFP-NLS
CSB
KHDRBS1
EMD
POLR2E
POLR2B
POLR2A
DDB1
POLR2C
POLR2H
POLR2D
CSA
CUL4A
POLR2G
PARP1
EYA4
RBMX
POLR2J3
NHP2L1
CUL4B
b
CSBΔCIM vs GFP-NLS
Significance (-Log p)
Enrichment (log2)
GFP-CSBΔCIM
GFP-NLS
CSB
KHDRBS1
NHP2L1
POLR2E
PARP1
POLR2B
POLR2C
POLR2A
POLR2D
CUL4A
POLR2H
RBMX
POLR2G
CUL4B
CSA
EYA4
DDB1
EMD
POLR2J3
c
CSB WT vs CSBΔCIM
Significance (-Log p)
Enrichment (log2)
GFP-CSB WT
GFP-CSBΔCIM
CSB
POLR2G
DDB1
POLR2B
POLR2E
POLR2H
POLR2C
POLR2A
CSA
CUL4B
POLR2J3
POLR2D
CUL4A
NHP2L1
RBMX
EYA4
KHDRBS1
d
CRL4CSA
DDB1
CUL4A
CSA
RBX1
170
130
95
72
56
43
34
26
17
11
xlCSB
hsCSB
WT
ΔCIM
WT
ΔCIM
CSB-FLAG
250
150
100
75
50
37
25
20
15
10
e
CSB-KO
+ GFP-CSB
UV-C
Input
IP: RNAPIIo
WT
ΔCIM
WT
ΔCIM
RNAPIIo
CSB
TFIIH (p89)

Supplementary Figure 6. Mass-spectrometry after GFP-CSB pull-down, and purification of proteins. (a-c) Volcano plots depicting mass spectrometry analysis after GFP immunoprecipitation on the soluble and chromatin fraction of UV-irradiated (20 J/m^2) cells comparing (a) GFP-CSB^{WT} with GFP-NLS (b) GFP-$CSB^{\Delta CIM}$ with GFP-NLS, and (c) GFP-CSB^{WT} with GFP-$CSB^{\Delta CIM}$. The enrichment (log^2) is plotted on the x-axis and the significance (-log p-value) is plotted on the y-axis. The -log p-value threshold was set to 1.3 ($p<0.05$). The enrichment threshold was set to 1 and all significant hits are shown in green. Several selected hits are shown in red, and GFP-CSB is shown in blue (Links to interactive volcano plots: CSB WT vs GFP-NLS / CSBΔCIM vs GFP-NLS / CSB WT vs CSBΔCIM). (d) Coomassie gels of recombinant $xlCRL4^{CSA}$ complex and xlCSB or hsCSB variants. DDB1, CUL4A, and all CSB proteins contained an N-terminal FLAG-tag, and CSA contained a C-terminal Strep-tag II. (e) Endogenous RNAPII Co-IP in CSB-KO + GFP-CSB^{WT} and CSB-KO + GFP-$CSB^{\Delta CIM}$. At least two independent replicates of each IP experiment were performed obtaining similar results. Source data are provided as a Source Data file.

a

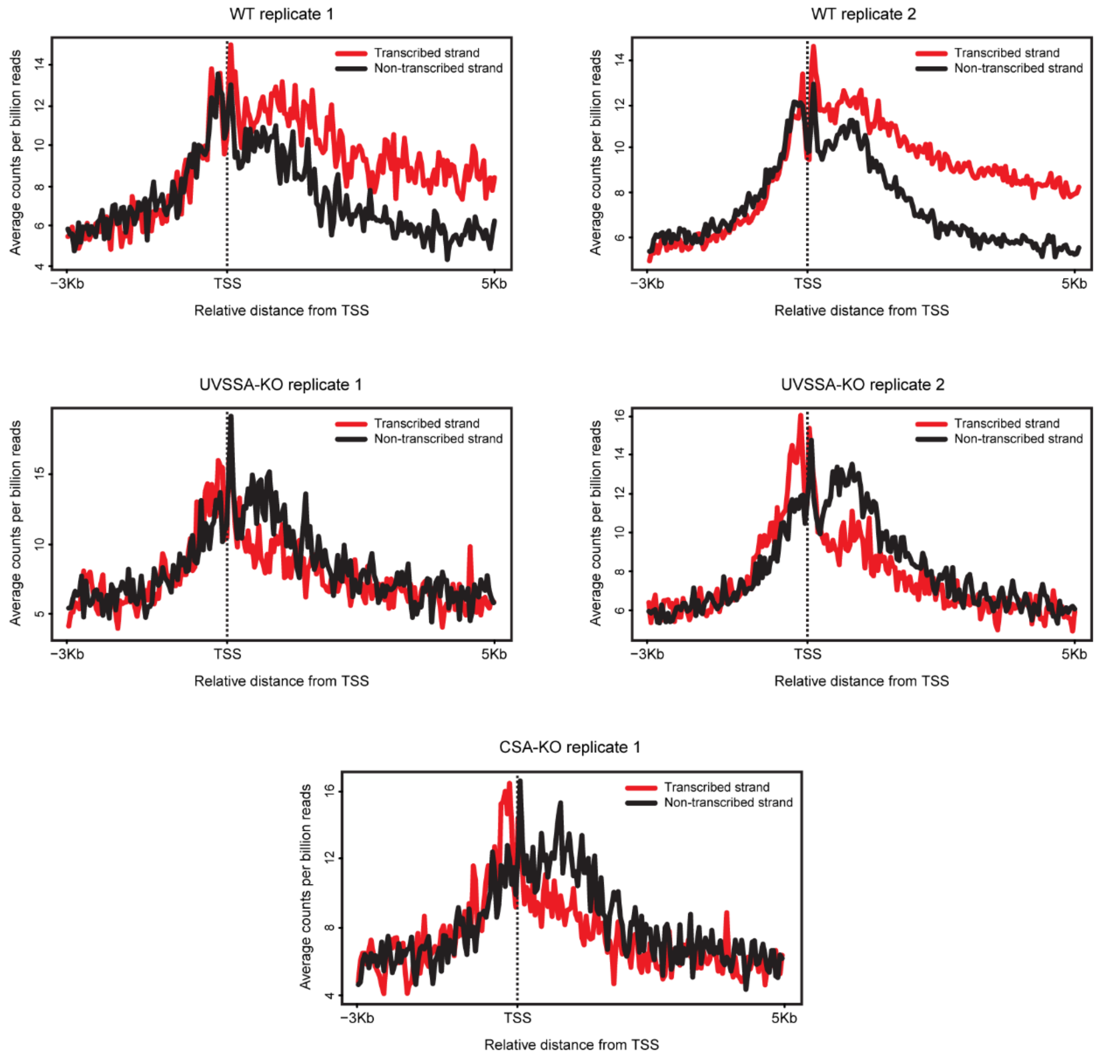
WT replicate 1
WT replicate 2
UVSSA-KO replicate 1
UVSSA-KO replicate 2
CSA-KO replicate 1
Transcribed strand
Non-transcribed strand
Average counts per billion reads
−3Kb
TSS
5Kb
Relative distance from TSS

b

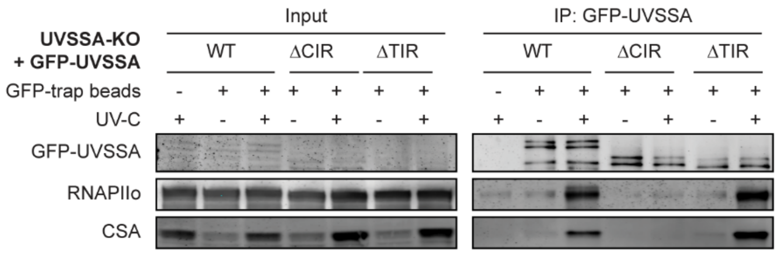
Input
IP: GFP-UVSSA
UVSSA-KO
+ GFP-UVSSA
WT
ΔCIR
ΔTIR
GFP-trap beads
UV-C
GFP-UVSSA
RNAPIIo
CSA

c

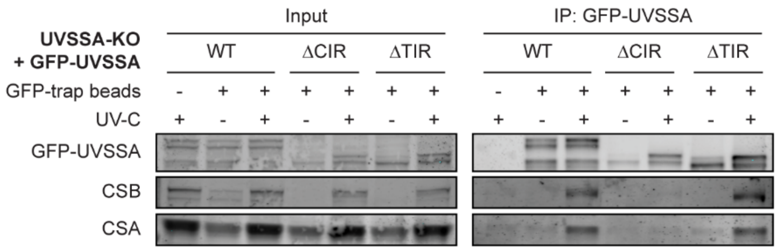
Input
IP: GFP-UVSSA
UVSSA-KO
+ GFP-UVSSA
WT
ΔCIR
ΔTIR
GFP-trap beads
UV-C
GFP-UVSSA
CSB
CSA

Supplementary Figure 7. XR-seq in TCR-KO cells, and immunoprecipitation in UVSSA mutants. (a) Average CPD XR-seq repair signal 3 Kb upstream and 5 Kb downstream of the annotated TSS of 16.088 genes in two independent biological replicates of experiments in WT and UVSSA-KO cells, and in a single replicate of CSA-KO cells. Signal is plotted separately for the transcribed (red) and non-transcribed (black) strands. The bin size of 40 nt. (b-c) Co-IP of GFP-UVSSA^{WT}, GFP-UVSSA$^{\Delta CIR}$, and GFP-UVSSA$^{\Delta TIR}$. At least two independent replicates of each IP experiment were performed obtaining similar results. Source data are provided as a Source Data file.

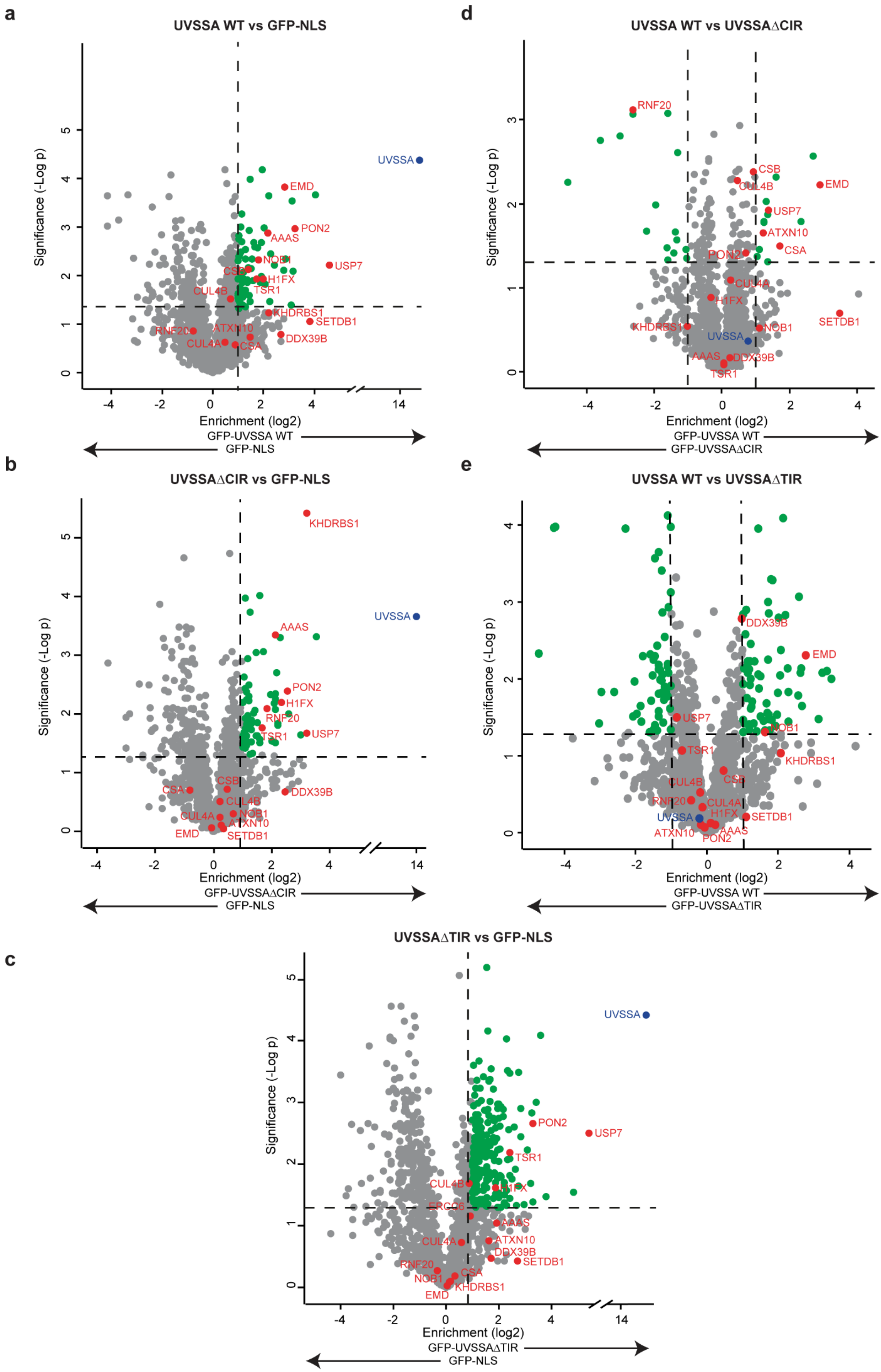
a
UVSSA WT vs GFP-NLS
Significance (-Log p)
Enrichment (log2)
GFP-UVSSA WT
GFP-NLS
UVSSA
EMD
PON2
AAAS
NOB1
USP7
CSB
H1FX
TSR1
CUL4B
KHDRBS1
SETDB1
RNF20
ATXN10
DDX39B
CUL4A
CSA
d
UVSSA WT vs UVSSAΔCIR
RNF20
CSB
CUL4B
EMD
USP7
ATXN10
CSA
PON2
CUL4A
H1FX
KHDRBS1
NOB1
SETDB1
UVSSA
AAAS
DDX39B
TSR1
GFP-UVSSAΔCIR
b
UVSSAΔCIR vs GFP-NLS
KHDRBS1
UVSSA
AAAS
PON2
H1FX
RNF20
USP7
TSR1
CSB
CSA
CUL4B
NOB1
CUL4A
ATXN10
EMD
SETDB1
DDX39B
e
UVSSA WT vs UVSSAΔTIR
DDX39B
EMD
USP7
NOB1
TSR1
KHDRBS1
CSB
CUL4B
RNF20
CUL4A
H1FX
UVSSA
SETDB1
ATXN10
AAAS
PON2
GFP-UVSSAΔTIR
c
UVSSAΔTIR vs GFP-NLS
UVSSA
PON2
USP7
TSR1
CUL4B
H1FX
ERCC6
AAAS
CUL4A
ATXN10
DDX39B
SETDB1
RNF20
NOB1
CSA
KHDRBS1
EMD

Supplementary Figure 8. Mass-spectrometry after GFP-UVSSA pull-down. (a-e) Volcano plots depicting mass spectrometry analysis after GFP immunoprecipitation on the soluble and chromatin fraction of UV-irradiated (20 J/m^2) cells comparing (a) GFP-UVSSA^{WT} with GFP-NLS (b) GFP-UVSSA$^{\Delta CIR}$ with GFP-NLS, (c) GFP-UVSSA$^{\Delta TIR}$ with GFP-NLS, (d) GFP-UVSSA^{WT} with GFP-UVSSA$^{\Delta CIR}$, and (e) GFP-UVSSA^{WT} with GFP-UVSSA$^{\Delta TIR}$. The enrichment (log^2) is plotted on the x-axis and the significance (-log p-value) is plotted on the y-axis. The -log p-value threshold was set to 1.3 ($p<0.05$). The enrichment threshold was set to 1 and all significant hits are shown in green. Several selected hits are shown in red, and GFP-UVSSA is shown in blue (Links to interactive volcano plots: UVSSA WT vs GFP-NLS / UVSSAΔCIR vs GFP-NLS / UVSSAΔTIR vs GFP-NLS / UVSSA WT vs UVSSAΔCIR / UVSSA WT vs UVSSAΔTIR).

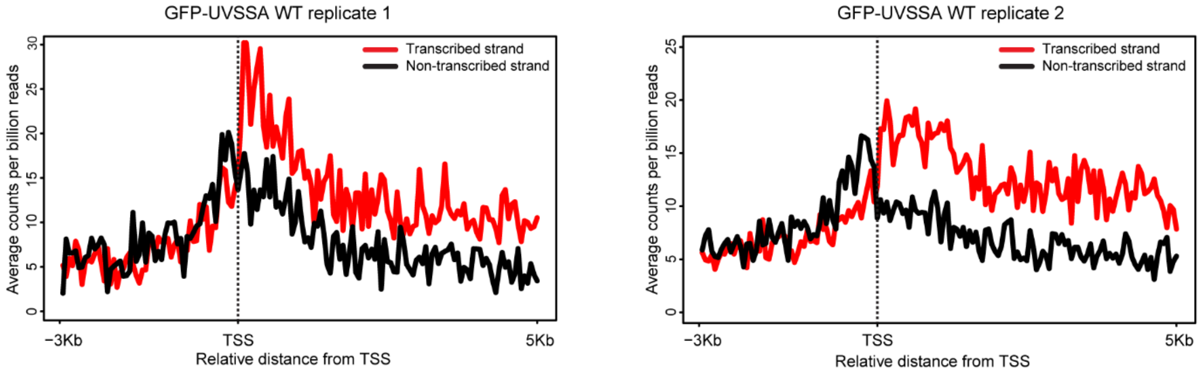

GFP-UVSSA WT replicate 1
Transcribed strand
Non-transcribed strand
Average counts per billion reads
0
5
10
15
20
25
30
−3Kb
TSS
5Kb
Relative distance from TSS
GFP-UVSSA WT replicate 2
Transcribed strand
Non-transcribed strand
Average counts per billion reads
0
5
10
15
20
25
−3Kb
TSS
5Kb
Relative distance from TSS

Supplementary Figure 9. XR-seq in GFP-UVSSA WT cells. Average CPD XR-seq repair signal 3 Kb upstream and 5 Kb downstream of the annotated TSS of 16,088 genes in two independent biological replicates of experiments in GFP-UVSSA WT cells. Signal is plotted separately for the transcribed (red) and non-transcribed (black) strands, with a bin size of 53 nt.

Supplementary Table 1: Cell lines

Cell lines	Origin
KPS3-hTERT	[1]
KPS3-hTERT + UVSSA	[1]
Sf9	Expression Systems (cat # 94-001S)
U2OS (FRT)	[2]
U2OS (FRT) CSA-KO (2-4)	This study
U2OS (FRT) CSA-KO (2-4) + CSA-GFP-5	This study
U2OS (FRT) CSB-KO (1-12)	This study
U2OS (FRT) CSB-KO (1-12) + GFP-CSB∆CIM-4	This study
U2OS (FRT) CSB-KO (1-12) + GFP-CSB-3	This study
U2OS (FRT) UVSSA-KO (1-8)	This study
U2OS (FRT) UVSSA-KO (1-8) / CSA (2-40) + GFP-UVSSA-3	This study
U2OS (FRT) UVSSA-KO (1-8) / CSB-KO (1-12) + GFP-UVSSA-3	This study
U2OS (FRT) UVSSA-KO (1-8) + GFP-UVSSA∆CIR-1	This study
U2OS (FRT) UVSSA-KO (1-8) + GFP-UVSSA∆TIR-6	This study
U2OS (FRT) UVSSA-KO (1-8) + GFP-UVSSA-3	This study
U2OS (FRT) XPA-KO (2-8)	This study
U2OS (FRT) XPC-KO (2-7)	This study
U2OS 2-6-3	[3]

Supplementary Table 2: Plasmids

Plasmids	Origin
pcDNA5/FRT/TO-Neo	Addgene #41000
pcDNA5/FRT/TO-Puro	This study
pcDNA5/FRT/TO-Puro-CSA^{WT}-GFP	This study
pcDNA5/FRT/TO-Puro-GFP-C1	This study
pcDNA5/FRT/TO-Puro-GFP-CSB$^{\Delta CIM}$	This study
pcDNA5/FRT/TO-Puro-GFP-CSB^{WT}	This study
pcDNA5/FRT/TO-Puro-GFP-N1	This study
pcDNA5/FRT/TO-Puro-GFP-UVSSA$^{\Delta CIR}$	This study
pcDNA5/FRT/TO-Puro-GFP-UVSSA$^{\Delta TIR}$	This study
pcDNA5/FRT/TO-Puro-GFP-UVSSA^{WT}	This study
pEGFP-C1	Clontech
pEGFP-N1	Clontech
pLV-U6g-PPB	LUMC/Sigma-Aldrich sgRNA library
pmCherry-LacR- UVSSA$^{\Delta TIR}$	This study
pmCherry-LacR-C1	[4]
pmCherry-LacR-C3	This study
pmCherry-LacR-CSB^{N}	This study
pmCherry-LacR-CSB^{M}	This study
pmCherry-LacR-CSB^{C}	This study
pmCherry-LacR-CSB$^{\Delta N}$	This study
pmCherry-LacR-CSB$^{\Delta C}$	This study
pmCherry-LacR-CSB$^{1221\text{-}1305}$	This study
pmCherry-LacR-CSB$^{1400\text{-}1493}$	This study
pmCherry-LacR-CSB$^{1417\text{-}1493}$	This study
pmCherry-LacR-CSB$^{1306\text{-}1399}$	This study
pmCherry-LacR-CSB$^{\Delta 1306\text{-}1300}$	This study
pmCherry-LacR-CSB$^{\Delta 1306\text{-}1352}$	This study
pmCherry-LacR-CSB$^{\Delta 1353\text{-}1399}$	This study
pmCherry-LacR-CSB$^{\Delta 1400\text{-}1428}$	This study
pmCherry-LacR-CSB$^{\Delta 1353\text{-}1368}$	This study
pmCherry-LacR-CSB$^{\Delta 1369\text{-}1384}$	This study
pmCherry-LacR-CSB$^{\Delta 1385\text{-}1399}$	This study
pmCherry-LacR-CSB^{WT}	This study
pmCherry-LacR-NLS	[5]
pmCherry-LacR-UVSSA$^{\Delta CIR}$	This study
pmCherry-LacR-UVSSA^{WT}	This study
pOG44	Thermo Fisher
pX458	Addgene #48138
pTM58_pAB1_FLAG-xlDDB1_x_(pIDC_xlCSA-StrepII)x2	This study
pTM65_pAB1_FLAG-xlCSB^{WT}	This study
pTM67_pAB1_FLAG-xlCUL4A_xlRBX1	This study
pTM141_pAB1_FLAG-xlCSB$^{\Delta CIM}$	This study
pTM142_pAB1_FLAG-hsCSB^{WT}	This study
pTM143_pAB1_FLAG-hsCSB$^{\Delta CIM}$	This study

Supplementary Table 3: Sequences of sgRNAs

sgRNAs		
CSB/ERCC6	5-AGACAGAATGATCCGATGAGGGG-3	sgML#003
CSA/ERCC8	5-CCAGACTTCAAGTCACAAAGTTG-3	sgML#018
UVSSA	5-AGAGAGCTGCTTTAGGCTGCTGG-3	sgML#019
XPA	5-CCTGTGTCAATTATCTTTGGGGC-3	sgML#002
XPC	5-TGGGGGTTTCTCATCTTCAAAGG-3	sgML#014

Supplementary Table 4: Sequencing primers to validate KO cell lines

Sequencing primers for knockouts		
CSB/ERCC6	5-GTAGGGGCCAGTTGTTAGAATGTAA-3	oML#078_sgML#003_CSB1_fw
	5-CTCACATTCTGAATGACTTGGCTA-3	oML#079_sgML#003_CSB1_rev
CSA/ERCC8	5-CAGTCTGTGTCCAGTTTCTGTG-3	oML#084_sgML#018_CSA_2FW
	5-CATATTTGTTATGTGTTTCTTTGAG-3	oML#085_sgML#018_CSA_2RV
	5-GTACATACATACATACACATTTACCAATAC-3	oML#100_sgML#018_CSA_2_Fw_Seq
	5-CTGAGAAAAAATGTACCTAAATATTAAG-3	oML#101_sgML#018_CSA_2_Rv_Seq
UVSSA	5-ACCCAGAGGTACACAGAGATTG-3	oML#090_sgML#019_UVSSA1_Fw
	5-GCTCTTAGAAGTGTCCCTGTG-3	oML#091_sgML#019_UVSSA1_Rv
	5-ATCAGGAGGCTGAGGCGGCTG-3	oML#076_sgML#020_UVSSA2_fw
	5-AGGAGCCTACCCGGGAGCCGGG-3	oML#077_sgML#020_UVSSA2_rev

Supplementary Table 5: Primers

Primers		
CSB WT	TTAAGTCGACCCAAATGAGGGAATCCCCCAC	oML#375
	AATTGCGGCCGCTTAGCAGTATTCTGGCTTGAGTTTC	oML#376
CSA WT	CACAATGCTAGCGCCACCATGCTGGGGTTTTTGTCCG	oML#041
	GCATGGTGAACTACCGGTGCTCCTTCTTCATCACTGCTG	oML#042
UVSSA WT	ACAATTGAATTCGATGGATCAGAAACTTTCGAAG	oML#035
	GTGTAAAGATCTCTAGTTCAGTGCGTAGTTAAAC	oML#036
CSBΔC	TCCAGCCTCGAGGTCCAAATGAGGGAATCCCCCACTC	oML#173
	TCAGGTCGGATCCTTATCGAGTTCCTTCAAACTTGGCGTCTC	oML#174
CSB-N	TCCAGCCTCGAGGTCCAAATGAGGGAATCCCCCACTC	oML#173
	GCATCAGGTCGGATCCTTAATCTCCATCATCTCGGTATCTTCCCAC	oML#178
CSBΔN	TCCAGCCTCGAGGTGATGGAGATGAAGATTATTATAAGCAGCGG	oML#175
	GCATCAGGTCGGATCCTTAGCAGTATTCTGGCTTGAGTTTCCAAATTC	oML#176
CSB-M	TCCAGCCTCGAGGTGATGGAGATGAAGATTATTATAAGCAGCGG	oML#175
	TCAGGTCGGATCCTTATCGAGTTCCTTCAAACTTGGCGTCTC	oML#174
CSB-C	TCCAGCCTCGAGGTCGAATTCCACACCTGGTGAAGAAAAG	oML#177
	GCATCAGGTCGGATCCTTAGCAGTATTCTGGCTTGAGTTTCCAAATTC	oML#176
CSB 1221-1305	TCCAGCCTCGAGGTCGAATTCCACACCTGGTGAAGAAAAG	oML#177
	GATGGAGGATCCTTACAGACACCGCTGACGAGAGAG	oML#196
CSB 1306-1399	TACAGCCTCGAGGTGGAGCAGTGTCTGGTGTTCCC	oML#197
	GGCGATGGAGGATCCTTACAGGTGGTTTCTAGCTCTCATTTTAGC	oML#198
CSB 1400-1493	TCCAGCCTCGAGGTATTCTGCCAGAGCGTTTAGAAAGTGAAAG	oML#199
	GCATCAGGTCGGATCCTTAGCAGTATTCTGGCTTGAGTTTCCAAATTC	oML#176
CSB 1417-1493	TACATCCTCGAGGTGCCCTGCTGCCCACCACAG	oML#200
	GCATCAGGTCGGATCCTTAGCAGTATTCTGGCTTGAGTTTCCAAATTC	oML#176
CSB Δ1306-1352	GCTCTCTCGTCAGCGGTGTCTGTGCCAGGATGGCATCATGAA	oML#226
	CCTTTTTCATGATGCCATCCTGGCACAGACACCGCTGACGAG	oML#227
CSB Δ1353-1399	CCTTCATCAACATCTCCAACAGAGAAGATTCTGCCAGAGCGTTTAG	oML#232
	CACTTTCTAAACGCTCTGGCAGAATCTTCTCTGTTGGAGATGTTG	oML#233
CSB Δ1306-1399	GAGGCTCTCTCGTCAGCGGTGTCTGATTCTGCCAGAGCGTTTAGAAAGTG	oML#224
	CTTTCACTTTCTAAACGCTCTGGCAGAATCAGACACCGCTGACGAGAG	oML#225
CSB Δ1400-1428	GCTAAAATGAGAGCTAGAAACCACCTGGTGGAGATGAGAAACTTCATC	oML#234
	GAAAGCGATGAAGTTTCTCATCTCCACCAGGTGGTTTCTAGCTCTC	oML#235
CSB Δ1353-1368	CCTTCATCAACATCTCCAACAGAGAAGCATTTTAGTGGAAGAGCAGAAG	oML#262
	CTGCATCTTCTGCTCTTCCACTAAAATGCTTCTCTGTTGGAGATGTTGA	oML#263
CSB Δ1369-1384	GAAAAAGGAGGGAAAAGATAATGTCCCTGAGGCTTCCTCCTCACTCTTG	oML#264
	CATTTTAGCCAAGAGTGAGGAGGAAGCCTCAGGGACATTATCTTTTCC	oML#265
CSB Δ1385-1399	AGACTCTTCATCCGGGCCCCTCATTCTGCCAGAGCGTTTAGA	oML#266
	CTTTCACTTTCTAAACGCTCTGGCAGAATGAGGGGCCCGGATGA	oML#267
UVSSA Δ100-200	CACAGACCCCGCACAGCCTCTGAGGCTGCTGGTGCCTTTTG	oML#128
	CAAAGTCAAAAGGCACCAGCAGCCTCAGAGGCTGTGCGGGG	oML#129
UVSSA Δ400-500	GGACAGAAGCCCTGGGGGATGCGGTGGTGCCCTACGGCGTG	oML#138
LacR-C3	ATTAAAACGCGTCAGTGGGCTGATC	oML#377
	TAATAATAGATCTGAAACCTTCCTCTTCTTCTTAG	oML#378

Supplementary Table 6: Antibodies

Antibodies	Host		Clone	WB	
Cas9	Mouse	Cell Signalling technology, #14697	7A9 and 3A3	1/5000	aML#031
CDK7	Mouse	kindly provided by J.M. Egly	2F8	1/2000	aML#076
CDK9	Rabbit	Bethyl	A303-493A	1/2000	aML#109
CPD	mouse	Cosmo Bio, CAC-NM-DND-001	TDM-2	1/1000	aML#020
CSA/ERCC8	Mouse	Santa Cruz, sc-376981	D2	1/500	aML#025
CSA/ERCC8	Rabbit	Abcam, 137033	EPR9237	1/750	aML#028
CSB/ERCC6	Goat	Santa Cruz, SC-10459	E-18	1/1000	aML#039
CSB/ERCC6	Rabbit	Santa Cruz, sc25370	H-300	1/300	aML#003
CUL4	Mouse	Santa Cruz, sc-377188	H11	1/100	aML#128
DDB1	Goat	Abcam, ab9194		1/1000	aML#035
ERCC1	Mouse	Santa Cruz, sc-17809	D10	1/300	aML#066
FLAG	Rabbit	New England Peptide; antigen: C(dPEG4)DYKDDDDK		1/5000	
GFP	Mouse	Roche, #11814460001	7.1 and 13.1	1/1000	aML#011
GFP	Rabbit	Abcam, ab290		1/1000	aML#044
Goat IgG (H+L) CF680	Donkey	Thermo fisher Scientific, A21084		1/10000	aML#037
Mouse IgG (H+L) CF770	Goat	Biotium, VWR #20077		1/10000	aML#009
Mouse IgG (HRP)	Goat	Abcam, ab6789		1/10000	aML#132
p44/ GTF2H2	Mouse	kindly provided by J.M. Egly	1H5	1/2000	aML#075
p62/GTF2H1	Mouse	kindly provided by J.M. Egly	3C9	1/2000	aML#074
p62/GTF2H1	Mouse	Santa Cruz, sc-48431	G10	1/500	aML#099
p62/GTF2H1	Mouse	Santa Cruz, sc25329	H10		
p80/XPD/ ERCC2	Mouse	Abcam, ab54676		1/500	aML#029
p89/XPB/ERCC3	Mouse	Millipore, MABE1123	15TF2-1B3	1/2000	aML#101
p89/XPB/ERCC3	Mouse	kindly provided by J.M. Egly	1B3	1/1000	aML#073
p89/XPB/ERCC3	Rabbit	Santa Cruz, SC-293	S-19	1/1000	aML#040
p89/XPB/ERCC3	Mouse	Santa Cruz, sc271500	G10		
rabbit IgG (H+L) CF680	Goat	Biotium, VWR #20067		1/10000	aML#010
Rbx1	Mouse	Santa Cruz, sc-393640	E11	1/100	aML#129
RNAPII-S2	Rabbit	Abcam, ab5095		1/1000	aML#024
RNAPII-S5	Mouse	Abcam, ab5408	4H8	1/1000	aML#125
Tubulin	Mouse	Sigma, T6199	DM1A	1/1000	aML#008
UVSSA	Mouse	Genetex, GTX629742	GT816	1/500	aML#100
UVSSA	Rabbit	Novus Biologicals, NBP1-32598		1/1000	aML#030

UVSSA	Rabbit	Abcam ab137644		1/1000	aML#034
UVSSA	Rabbit	Genetex, GTX106751		1/1000	aML#087
xlCSA	Rabbit	New England Peptide; antigen: CHRTHINPAFEDAWSSSEDES		1/5000	
XPA	Rabbit	kindly provided by Rick Wood	CJ1	1/10000	aML#079
XPC	Rabbit	Novus Biologicals, NB100-58801		1/2000	aML#077
XPF/ ERCC4	Mouse	Santa Cruz, sc-136153	3F2/3	1/200	aML#096

Supplementary Table 7: Link to interactive volcano plots

Figure	Link
S6a	CSB WT vs GFP-NLS
S6b	CSBΔCIM vs GFP-NLS
S6c	CSB WT vs CSBΔCIM
5b	GFP-UVSSA vs GFP-UVSSA +UV
S8a	UVSSA WT vs GFP-NLS
S8b	UVSSAΔCIR vs GFP-NLS
S8c	UVSSAΔTIR vs GFP-NLS
S8d	UVSSA WT vs UVSSAΔCIR
S8e	UVSSA WT vs UVSSAΔTIR

Supplementary Table 8: Sequence depth of individual XR-seq replicates

Cell lines	Replicate	Reads
U2OS (FRT) WT	1	643,044
U2OS (FRT) WT	2	413,598
U2OS (FRT) UVSSA-KO (1-8)	1	254,272
U2OS (FRT) UVSSA-KO (1-8)	2	1,061,527
U2OS (FRT) CSA-KO (2-4)	1	309,814
U2OS (FRT) UVSSA-KO (1-8) + GFP-UVSSA-3	1	61,660
U2OS (FRT) UVSSA-KO (1-8) + GFP-UVSSA-3	2	134,133
U2OS (FRT) UVSSA-KO (1-8) + GFP-UVSSAΔCIR-1	1	266,029
U2OS (FRT) UVSSA-KO (1-8) + GFP-UVSSAΔTIR-6	1	527,179

Supplementary References

1. Nakazawa, Y. *et al.* Mutations in UVSSA cause UV-sensitive syndrome and impair RNA polymerase IIo processing in transcription-coupled nucleotide-excision repair. *Nat Genet* **44**, 586-592 (2012).
2. Panier, S. *et al.* Tandem protein interaction modules organize the ubiquitin-dependent response to DNA double-strand breaks. *Mol Cell* **47**, 383-395 (2012).
3. Janicki, S.M. *et al.* From silencing to gene expression: real-time analysis in single cells. *Cell* **116**, 683-698 (2004).
4. Luijsterburg, M.S. *et al.* A PALB2-interacting domain in RNF168 couples homologous recombination to DNA break-induced chromatin ubiquitylation. *eLife* **6** (2017).
5. Soutoglou, E. & Misteli, T. Activation of the cellular DNA damage response in the absence of DNA lesions. *Science* **320**, 1507-1510 (2008).
