# Supplementary material for: The cooperative action of CSB, CSA, and UVSSA target TFIIH to DNA damage-stalled RNA polymerase II
Source: Nat Commun. 2020 Apr 30;11:2104. doi: 10.1038/s41467-020-15903-8 (PMC7192910; doi:10.1038/s41467-020-15903-8)
Supplement: Supplementary file 3 — Reporting Summary [file 41467_2020_15903_MOESM3_ESM.pdf]

nature research

Corresponding author(s): M.S. Luijsterburg

Last updated by author(s): Mar 19, 2020

Reporting Summary

Nature Research wishes to improve the reproducibility of the work that we publish. This form provides structure for consistency and transparency in reporting. For further information on Nature Research policies, see Authors & Referees and the Editorial Policy Checklist.

Statistics

For all statistical analyses, confirm that the following items are present in the figure legend, table legend, main text, or Methods section.

n/a	Confirmed	
☐	☒	The exact sample size (*n*) for each experimental group/condition, given as a discrete number and unit of measurement
☐	☒	A statement on whether measurements were taken from distinct samples or whether the same sample was measured repeatedly
☐	☒	The statistical test(s) used AND whether they are one- or two-sided<br>*Only common tests should be described solely by name; describe more complex techniques in the Methods section.*
☒	☐	A description of all covariates tested
☒	☐	A description of any assumptions or corrections, such as tests of normality and adjustment for multiple comparisons
☐	☒	A full description of the statistical parameters including central tendency (e.g. means) or other basic estimates (e.g. regression coefficient) AND variation (e.g. standard deviation) or associated estimates of uncertainty (e.g. confidence intervals)
☒	☐	For null hypothesis testing, the test statistic (e.g. *F*, *t*, *r*) with confidence intervals, effect sizes, degrees of freedom and *P* value noted<br>*Give P values as exact values whenever suitable.*
☒	☐	For Bayesian analysis, information on the choice of priors and Markov chain Monte Carlo settings
☒	☐	For hierarchical and complex designs, identification of the appropriate level for tests and full reporting of outcomes
☒	☐	Estimates of effect sizes (e.g. Cohen's *d*, Pearson's *r*), indicating how they were calculated

Our web collection on statistics for biologists contains articles on many of the points above.

Software and code

Policy information about availability of computer code

Data collection	All software used in this study has been described in published literature and are detailed in the method section of the manuscript.
Data analysis	Mass spectrometry data was analyzed using MaxQuant software (v1.5.3.30) and analysis output was further processed in the Perseus (v 1.5.5.3) computational platform. Microscopy images were acquired using ZEN 2012 software (blue edition, version 1.1.0.0). Adapter sequence was trimmed from each read using Trimmomatic (version 0.36). Reads were aligned to the genome using Bowtie (v 1.2.2). XR-seq data was analzed using bedtools (v2.26.0), PicardCommandLine MarkDuplicates (v 2.8.1), R (Version 3.5.1), genomation package of Bioconductor(v 1.14.0). R/Shiny was used to make the web app VolcaNoseR (V 1.0.0) for interactive volcanos.

For manuscripts utilizing custom algorithms or software that are central to the research but not yet described in published literature, software must be made available to editors/reviewers. We strongly encourage code deposition in a community repository (e.g. GitHub). See the Nature Research guidelines for submitting code & software for further information.

Data

Policy information about availability of data

All manuscripts must include a data availability statement. This statement should provide the following information, where applicable:
- Accession codes, unique identifiers, or web links for publicly available datasets
- A list of figures that have associated raw data
- A description of any restrictions on data availability

Mass spectrometry proteomics data are presented in main Fig 5b and Supplementary Fig 6a-c and 8a-e, and have been deposited to the ProteomeXchange Consortium via the PRIDE partner repository61 (https://www.ebi.ac.uk/pride/) with the dataset identifiers PXD013572 and PXD017329. Processed mass spectrometry proteomics data used for interactive Volcano plots is deposited on Zenodo.org (DOI: 10.5281/zenodo.3713174). The code for VolcaNoseR (Version 1.0.0) has been deposited on Zenodo.org (DOI: 10.5281/zenodo.3625858). XR-seq data are presented in main Fig 5f, 7b, and Supplementary Fig S7a, Fig S9a. XR-seq data are deposited in the Gene Expression Omnibus (GEO; https://www.ncbi.nlm.nih.gov/geo/) under GSE132840. Additional data and custom code will be made

available upon reasonable request. The source data underlying Figs 1b, d-h, 2d, 3b-g, 4a-d, 5a, c-e, 6c-f, and 7a and Supplementary Figs 1a-c, 2c, 3c, 4c, 6d-e and 7b-c are provided as a Source Data file.

Field-specific reporting

Please select the one below that is the best fit for your research. If you are not sure, read the appropriate sections before making your selection.

☒ Life sciences ☐ Behavioural & social sciences ☐ Ecological, evolutionary & environmental sciences

For a reference copy of the document with all sections, see nature.com/documents/nr-reporting-summary-flat.pdf

Life sciences study design

All studies must disclose on these points even when the disclosure is negative.

Sample size	The number of replicate experiments are indicated in the figure legends of the manuscript and at least two replicates were performed for each individual approach except for XR-seq. ample sizes were chosen for the differing experimental approaches based on the technical difficulty and throughput of the individual assays.
Data exclusions	No data was excluded
Replication	- Knockout cells were confirmed by sequencing and if specific antibodies were available also with western blot analyses<br>- Interactions found with the chromatin-tethering approach were confirmed with immunoprecipitation and western blot analyses<br>- Mass spec findings were confirmed by reciprocal immunoprecipitation and western blot analyses<br>- IP experiment were repeated 2 - 5 times. All attempts at reproduction were succesful. Mass spec was performed in quadruplicate. XR-seq was performed 1 - 2 times (indicated in the legends). LacR tethering experiments were performed 2- 8 times (indicated in the legends)
Randomization	Randomization was not applicable to our study. The assays used in this study are not subject to systematic variation that demands randomisation
Blinding	Blinding was not applicable to our study. Blinding in IP / western blot approaches is not possible.

Reporting for specific materials, systems and methods

We require information from authors about some types of materials, experimental systems and methods used in many studies. Here, indicate whether each material, system or method listed is relevant to your study. If you are not sure if a list item applies to your research, read the appropriate section before selecting a response.

Materials & experimental systems

n/a	Involved in the study	
☐	☒	Antibodies
☐	☒	Eukaryotic cell lines
☒	☐	Palaeontology
☒	☐	Animals and other organisms
☒	☐	Human research participants
☒	☐	Clinical data

Methods

n/a	Involved in the study	
☐	☒	ChIP-seq
☒	☐	Flow cytometry
☒	☐	MRI-based neuroimaging

Antibodies

Antibodies used	Cas9 (Mouse) - Cell Signalling technology (7A9-3A3 #14697); RRID:AB_2750916; Lot #2<br>CSA/ERCC8 (Mouse) - Santa Cruz (D2 sc-376981); Lot #10816<br>CSA/ERCC8 (Rabbit) - Abcam (EPR9237 ab137033); RRID:AB_2783825; Lot #GR155793-4<br>CSB/ERCC6 (Goat) - Santa Cruz (E-18 SC-10459); RRID:AB_668957; Lot #A2114<br>CSB/ERCC6 (Rabbit) - Santa Cruz (H-300 sc25370); RRID:AB_668958; Lot #I1012<br>DDB1 (Goat) - Abcam (ab9194); RRID:AB_307063; Lot #GR35765-27<br>ERCC1 (Mouse) - Santa Cruz (D10 sc-17809); Lot #H2316<br>FLAG (Rabbit) - New England Peptide; antigen: C(dPEG4)DYKDDDDK<br>GFP (Mouse) - Roche (7.1-13.1 11814460001); RRID:AB_390913; Lot #27575600<br>GFP (Rabbit) - Abcam (ab290); RRID:AB_303395; Lot #GR3251545-1<br>Goat IgG (H+L) CF680 (Donkey) - Thermo fisher Scientific (A-21084); RRID:AB_2535741; Lot #1778374<br>Mouse IgG (H+L) CF770 (Goat) - Biotium (#20077); RRID: AB_10852670; Lot #17C0928<br>p44/GTF2H2 (Mouse) kindly provided by J.M. Egly (1H5)<br>p62/GTF2H1 (Mouse) kindly provided by J.M. Egly (3C9)<br>p62/GTF2H1 (Mouse) - Santa Cruz (G10 sc-48431); RRID:AB_2247962; Lot #J1706<br>p80/XPD/ERCC2 (Mouse) - Abcam (ab54676); RRID:AB_946174; Lot #GR302689-I<br>p89/XPB/ERCC3 (Mouse) - Millipore (15TF2-1B3 MABE1123); Lot #Q2589558

p89/XPB/ERCC3 (Mouse) - kindly provided by J.M. Egly (1B3)
p89/XPB/ERCC3 (Rabbit) - Santa Cruz (S-19 SC-293); RRID:AB_2262177; Lot #J1912
rabbit IgG (H+L) CF680 (Goat) - Biotium (VWR #20067); RRID:AB_10871686; Lot #18C1120
RNAPII-S2 (Rabbit) - Abcam (ab5095); RRID:AB_304749; Lot #GR3231908-7
RNAPII-S5 (Mouse) - Abcam (4H8 ab5408); RRID:AB_304868; Lot #GR3264297-4
Tubulin (Mouse) - Sigma (DM1A T6199); RRID:AB_477583; Lot #048M4751V
UVSSA (Mouse) - Genetex (GT816 GTX629742); Lot #41428
UVSSA (Rabbit) - Novus Biologicals (NBP1-32598); RRID:AB_10003966; Lot #40002
UVSSA (Rabbit) - Abcam (ab137644); Lot #GR120730-1
UVSSA (Rabbit) - Genetex (GTX106751); RRID:AB_1950618; Lot #40002
xlCSA (Rabbit) - New England Peptide; antigen: CHRTHINPAFEDAWSSSEDES
XPA (Rabbit) - kindly provided by Rick Wood (CJ1)
XPC (Rabbit) - Novus Biologicals (NB100-58801); RRID:AB_877776; Lot #A1
XPF/ERCC4 (Mouse) - Santa Cruz (3F2/3 sc-136153); RRID:AB_2098034; Lot #G3014
CPD (Mouse) - Cosmo Bio (TDM-2 CAC-NM-DND-001); RRID:AB_1962813; Lot #TM-C-014
Mouse IgG (HRP) (goat) - Abcam (ab6789); RRID:AB_955439; Lot #GR3234199-2
CUL4 (Mouse) - Santa Cruz (H11 sc-377188); Lot #D0317
Rbx1 (Mouse) - Santa Cruz (E11 sc-393640); RRID:AB_2722527; Lot #A2618
CDK9 (Rabbit) - Bethyl (A303-493A); RRID:AB_10949230
CDK7 (Mouse) - kindly provided by J.M. Egly (2F8)
p62/GTF2H1 (Mouse) – Santa Cruz (H10, sc25329); RRID:AB_671295
p89/XPB/ERCC3 (Mouse) – Santa Cruz (G10, sc271500); RRID:AB_10649033

Validation

The following antibodies were validated in knockout cells: CSA/ERCC8 (Rabbit) - Abcam (EPR9237 ab137033), CSB/ERCC6 (Goat) - Santa Cruz (E-18 SC-10459), CSB/ERCC6 (Rabbit) - Santa Cruz (H-300 sc25370), XPA (Rabbit) - gift of Rick Wood (CJ1), XPC (Rabbit) Novus Biologicals, NB100-58801), UVSSA (Mouse) - Genetex (GT816 GTX629742), UVSSA (Rabbit) - Novus Biologicals (NBP1-32598), UVSSA (Rabbit) - Abcam (ab137644), UVSSA (Rabbit) - Genetex (GTX106751), XPF/ERCC4 (Mouse) - Santa Cruz (3F2/3 sc-136153), ERCC1 (Mouse) - Santa Cruz (D10 sc-17809)

The following antibodies were validated in Co-IP experiments: CSA/ERCC8 (Mouse) - Santa Cruz (D2 sc-376981), DDB1 (Goat) - Abcam (ab9194), p44/GTF2H2 (Mouse) kindly provided by J.M. Egly (1H5), p62/GTF2H1 (Mouse) kindly provided by J.M. Egly (3C9), p62/GTF2H1 (Mouse) - Santa Cruz (G10 sc-48431), p80/XPD/ERCC2 (Mouse) - Abcam (ab54676), p89 (Mouse) - Millipore (15TF2-1B3 MABE1123), p89/XPB/ERCC3 (Mouse) - kindly provided by J.M. Egly (1B3), p89/XPB/ERCC3 (Rabbit) - Santa Cruz (S-19 SC-293), RNAPII-S2 (Rabbit) - Abcam (ab5095), RNAPII-S5 (Mouse) - Abcam (4H8 ab5408), CUL4 (Mouse) - Santa Cruz (H11 sc-377188), Rbx1 (Mouse) - Santa Cruz (E11 sc-393640), CDK7 (Mouse) - kindly provided by J.M. Egly (2F8), FLAG (Rabbit) - New England Peptide; antigen: C(dPEG4)DYKDDDDK, xlCSA (Rabbit) - New England Peptide; antigen: CHRTHINPAFEDAWSSSEDES

The following antibody was validated in knock-down experiments: CDK9 (Rabbit) - Bethyl (A303-493A); Thales C. Nepomuceno. Et al. 2017

The following antibody is a commonly used loading control: Tubulin (Mouse) - Sigma (T6199)

The following antibody was validated by slot blot analysis: CPD (Mouse) - Cosmo Bio (TDM-2 CAC-NM-DND-001)

The following antibodies were validated by over-expression experiments: Cas9 (Mouse) - Cell Signalling technology (7A9-3A3 #14697), GFP (Mouse) - Roche (7.1-13.1 11814460001), GFP (Rabbit) - Abcam (ab290)

Eukaryotic cell lines

Policy information about cell lines

Cell line source(s)

KPS3-hTERT; Nakazawa, Y. et al. 2012
KPS3-hTERT + UVSSA; Nakazawa, Y. et al. 2012
U2OS (FRT); This study
U2OS (FRT) CSA-KO (2-4); This study
U2OS (FRT) CSA-KO (2-4) + CSA-GFP-5; This study
U2OS (FRT) CSB-KO (1-12); This study
U2OS (FRT) CSB-KO (1-12) + GFP-CSBDCIM-4; This study
U2OS (FRT) CSB-KO (1-12) + GFP-CSB-3; This study
U2OS (FRT) UVSSA-KO (1-8); This study
U2OS (FRT) UVSSA-KO (1-8) + GFP-UVSSADCIR-1; This study
U2OS (FRT) UVSSA-KO (1-8) + GFP-UVSSADTIR-6; This study
U2OS (FRT) UVSSA-KO (1-8) + GFP-UVSSA-3; This study
U2OS (FRT) UVSSA-KO (1-8) / CSA (2-40) + GFP-UVSSA-3; This study
U2OS (FRT) UVSSA-KO (1-8) / CSB-KO (1-12) + GFP-UVSSA-3; This study
U2OS (FRT) XPA-KO (2-8); This study
U2OS (FRT) XPC-KO (2-7); This study
U2OS 2-6-3; Janicki, S.M. et al. 2004
Sf9 cells; Expression Systems (cat # 94-001S)

Authentication

Cells were authenticated by STR profiling. All knockout cells were validated by Western blot analysis and DNA sequencing

Mycoplasma contamination	All cell lines were routinely tested for mycoplasma and were nested negative
Commonly misidentified lines (See ICLAC register)	No commonly misidentified cell lines were used in this study

ChIP-seq

Data deposition

☒ Confirm that both raw and final processed data have been deposited in a public database such as GEO.

☒ Confirm that you have deposited or provided access to graph files (e.g. BED files) for the called peaks.

Data access links
May remain private before publication.

Both raw and processed XR-seq data are deposited in the Gene Expression Omnibus (GEO; https://www.ncbi.nlm.nih.gov/geo/). The data can be accessed by using the following details:
Dataset identifier: GSE132840
Password: gjqtayoetdmjrov
Additional data will be provided upon request

Files in database submission

XR-seq Raw data: Inputs
CIR.fastq.gz
TIR.fastq.gz
WT_rep1.fastq.gz
WT_rep2.fastq.gz
CSA_1_TTAGGC_L005_R1.fastq.gz
U2OS_WT_1_CGATGT_L005_R1.fastq.gz
U2OS_WT_2_GATCAG_L005_R1.fastq.gz
UVSSA_1_TAGCTT_L005_R1.fastq.gz
UVSSAz_2_GGCTAC_L005_R1.fastq.gz

XR-seq Processed data (additional data will be made available upon request):
CIR___Minus_cov.bw
CIR___Plus_cov.bw
TIR___Minus_cov.bw
TIR___Plus_cov.bw
WT_rep1__Minus_cov.bw
WT_rep1__Plus_cov.bw
WT_rep2__Minus_cov.bw
WT_rep2__Plus_cov.bw
CSA_1_TTAGGC_Minus_cov.bw
CSA_1_TTAGGC_Plus_cov.bw
U2OS_WT_1_Minus_cov.bw
U2OS_WT_1_Plus_cov.bw
U2OS_WT_2_Minus_cov.bw
U2OS_WT_2_Plus_cov.bw
UVSSA_1_TAGCTT_Minus_cov.bw
UVSSA_1_TAGCTT_Plus_cov.bw
UVSSAz_2_GGCTAC_Minus_cov.bw
UVSSAz_2_GGCTAC_Plus_cov.bw

Genome browser session (e.g. UCSC)

no longer applicable

Methodology

Replicates

We performed CPD XR-seq in U2OS WT (n=2), UVSSA-KO (n=2), CSA-KO (n=1), GFP-UVSSA WT (n=2), GFP-UVSSA mutant (n=1) cells treated with UV irradiation (20J/m2).

Sequencing depth

All XR-seq samples were sequenced in signle-end with 50nt/75nt sequencing reads. Details on sequencing depth and uniquely mapped high quality reads are below.
XR-seq
Single-end 75 nt read length:
GFP-UVSSA-CIR mutant (n=1)
CIR.fastq: 12538114 reads. uniquely mapped reads: 266,029
GFP-UVSSA-TIR mutant (n=1)
TIR.fastq: 4,918,264 reads. uniquely mapped reads: 527,179
GFP-UVSSA WT (n=2)
WT_rep1.fastq: 4,467,975 reads. uniquely mapped reads: 61,489
WT_rep2.fastq: 5,253,802 reads. uniquely mapped reads: 134,133

Single-end 50 nt read length:
U2OS-WT (n=2)
U2OS_WT_1_CGATGT_L005_R1.fastq: 14,297,307 reads. uniquely mapped reads: 643,044
U2OS_WT_2_GATCAG_L005_R1.fastq: 16,707,019 reads. uniquely mapped reads: 4,135,989

UVSSA-KO (n=2)
UVSSA_1_TAGCTT_L005_R1.fastq: 14,618,138 reads. uniquely mapped reads: 254,272
UVSSAz_2_GGCTAC_L005_R1.fastq: 9,977,819 reads. uniquely mapped reads: 1,061,527

CSA-KO (n=2)
CSA_1_TTAGGC_L005_R1.fastq: 15,190,863 reads. uniquely mapped reads: 309,814

Antibodies	p62/GTF2H1 (Mouse) – Santa Cruz (H10, sc25329)<br>p89/XPB/ERCC3 (Mouse) – Santa Cruz (G10, sc271500)<br>CPD (Mouse) - Cosmo Bio (TDM-2 CAC-NM-DND-001); Lot #TM-C-014
Peak calling parameters	bowtie -q --nomaqround --phred33-quals -p 32 -m 4 -n 2 -e 70 -l 20 --chunkmbs 800 --best –S<br>Reads mapped to more than 4 positions were filtered using bowtie, allowing maximum 2 mismatches. Duplicate XR-seq reads were subsequently removed using PicardCommandLine MarkDuplicates.
Data quality	Data quality for individual replicates was confirmed by fastx-toolkit and FastQC checking read quality, level of duplications, GC-content, k-mer content and adapter contamination. TT enrichment in positions 19-21 was ensured after trimming adapter sequence from reads (specific positions of damage in excised oligomers).
Software	FastQC (v 0.11.7), Trimmomatic (v 0.36), Samtools (v 1.7), bowtie(v 1.2.2), bedtools (v2.26.0), PicardCommandLine MarkDuplicates (v 2.8.1), R (Version 3.5.1)
